# Hookworm and Pellagra

**Published:** 1910-01

**Authors:** W. A. Philpott

**Affiliations:** San Antonio


For Texas Medical Journal.

### Hookworm and Pellagra.

You may bid farewell to "itis,"
  Who has thrived so long on earth;
We will cut appendic-"itis,"
  Which has caused the surgeon mirth;
Bicillus and the typhoid germ,
  That's brought the doctors' smile—
For the naughty, naughty hookworm
  Is the fashion for a while!

You may have the chills and fever,
  Or the cholera morbus bad,
If your doc's a firm believer
  In the late pellagra fad,
Do not fear the pills and quinine,
  Though you are shaking with the ague,
He will will give you filtered "bile-ine"
  For the fatal corn meal plague!

If La Grippe holds tight its clutches—
  Scarcely breathe, nose running, red,
Bones so sore you wish for crutches;
  Loll about upon your bed;
Does dear doc say "influenza,"
  Then prescribe hot lemonade?
No—"the operating mensa,"
  As he sharps his hookworm blade!

Indigestion, phthisis, asthma,
  Smallpox, measles, whooping cough—
Are reduced to plain pellagra
  When you pull the "itis" off:
Cancer, Bright's disease, and tumor,
  And the dreaded tuber-germ,
When you analyze with humor,
  Just become the gay hookworm

—*W. A. Philpott, San Antonio.*

---

A young and apparently healthy man with tendo-synovitis should always be suspected of gonorrhea.—*American Journal of Surgery.*

